# Phylogeography and population structure of - *grypotus* (Richardson, 1846) as revealed by mitochondrial control region sequences

**DOI:** 10.3897/zookeys.705.13001

**Published:** 2017-10-03

**Authors:** Linlin Zhao, Dan Yi, Chunhou Li, Dianrong Sun, Hanxiang Xu, Tianxiang Gao

**Affiliations:** 1 The First Institute of Oceanography, SOA, Qingdao, Shandong, 266003, P.R. China; 2 South China Sea Fisheries Research Institute, Chinese Academy of Fishery Science, Guangzhou 510300, P.R. China; 3 Fishery College, Zhejiang Ocean University, Zhoushan, Zhejiang, 316000, P.R. China

**Keywords:** *Johnius grypotus*, mitochondrial control region, genetic diversity, genetic structure, population historical demography

## Abstract

The 137 individuals of *Johnius grypotus* were collected from seven localities from the Bohai Sea to the East China Sea. A 549 base pair (bp) fragment of the hypervariable region of the mtDNA control region was sequenced to examine genetic diversity and population structure. The populations of *J. grypotus* showed high haplotype diversity (*h*) with a range from 0.7500 to 0.9740 and low nucleotide diversity (*π*) with a range from 0.0024 to 0.0067. Low and non-significant genetic differentiation was estimated among populations except for North Yellow Sea population, which has a significant genetic difference with other populations. The demographic history examined by mismatch distribution analyses and Bayesian skyline plot (BSP) analyses revealed that a sudden population expansion occurred almost 20 to 40 thousand years before. Relatively recent population expansion in the last glacial period, large dispersal of eggs or larvae carried by coastal current, and the homogeneity of living environment may have an important influence on the population genetic pattern.

## Introduction

Among the many factors affecting the phylogeography and population structure of species, climate change has received the greatest attention (Avise, 2009). A vast body of evidence from the fossil and pollen records clearly demonstrates that many terrestrial species have undergone large and rapid latitudinal shifts in response to Pleistocene climate change, particularly following the end of the last glacial maximum (LGM), approximately 20000 years before present (Graham et al. 1996; Jim et al. 2008). Marine species show the same regulation, due to a series of large glacial–interglacial changes with sea level rise and fall in Pleistocene (Marko et al. 2010; Xavier et al. 2011). The living environment and the character of life history were also proved to have a crucial effect on the population genetic structure (Taylor et al. 2003; Hwang et al. 2005).

It is difficult to obtain fossil records in marine environment; therefore, the DNA makers play an important role to detect the phylogeography and population structure in marine species (Gary et al. 1998). The mitochondrial DNA (mtDNA) has been the marker of choice in studying population structure and inferring phylogenetic relationships in animals due to its strict maternal inheritance and absence of recombination in most species (Whitehead et al. 2003; Dowling et al. 2008). The mitochondrial genome is also variable as its mutation rate is 5-10 times faster than the nuclear genome (Castro et al. 1999). The most variable region of the mitochondrial genome is the mitochondrial control region, which mutates five times more rapidly than the rest of the genome (Brown et al. 1979). Therefore, it had been widely utilized as a genetic marker in detecting genetic diversity and population genetic structure of marine fishes (Avise et al. 1986; Liu et al. 2007; Galtier et al. 2009; Han et al. 2012; Song et al. 2013).

The family Sciaenidae in the order Perciformes is widely distributed throughout the world with approximately 70 genera and 300 species (Nelson 2006). The coastal area of China is one of the main distribution areas, which possesses about 30 species in 17 genera (Zhu et al. 1963). A member of this family, *Johnius grypotus*, is widely distributed along the China’s coastal sea, which lives in water areas with rocks or mud materials on bottom and never performs a habit of long distance migration (Zhu et al. 1963; Sasaki 1991). It is the tropical fish, which feeds on shrimps, shellfish, crabs, and small fish living in the bottom of the sea (Guo et al., 2017). The breeding season of *J. grypotus* is from June to September with planktonic larvae (Wang et al., 2012), and the duration of larval phase is about 30 days according to closely related species (Lei et al., 1981; Zhan et al., 2016). In the present study, mitochondrial control region sequences were used to investigate the phylogeography and population structure of *J. grypotus* that inhabits the coastal waters of the Bohai Sea, the Yellow Sea, and the East China Sea.

## Materials and methods

### Sample collection and DNA extration

**Figure 1.**
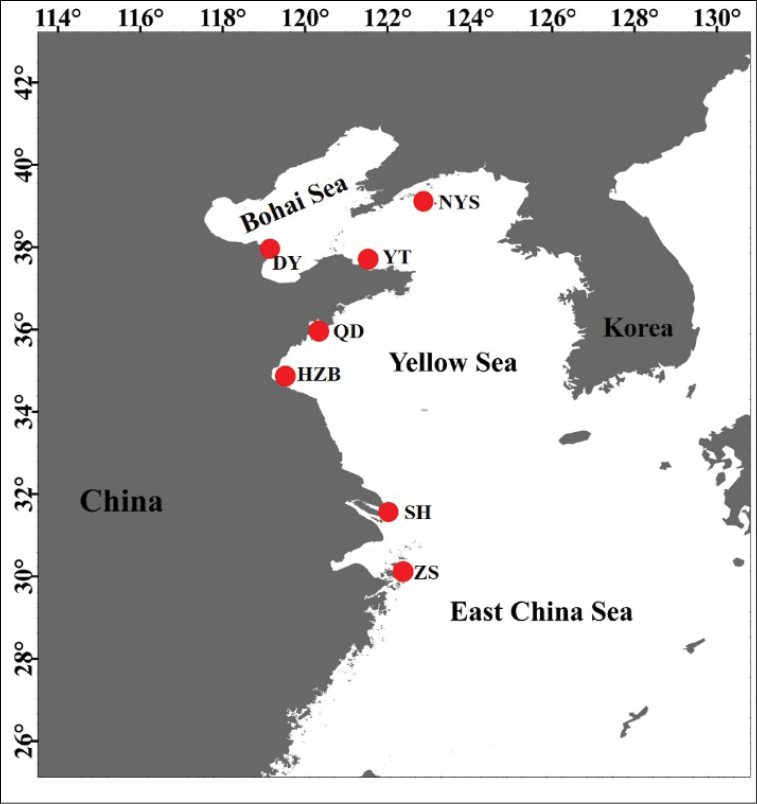
Sampling sites of *J. grypotus*

The specimens of *J. grypotus* were collected at seven locations from the Bohai Sea (DY, YT), the Yellow Sea (NYS, QD, HZB, SH) and the East China Sea (ZS) during 2005 to 2011 (Table 1, Fig. 1). Muscle tissues were preserved in 95% ethanol. Tissue samples were digested for two or three hours at 55°C in 200 μl extraction buffer containing 20 μl proteinase K. Genomic DNA was extracted following a standard phenol-chloroform method.

**Table 1.** Sample information and genetic diversity index of *J. grypotus*

Population	Sample code	Sampling date	Sample size	No. of polymorphic sites (*S*)	No. of Haplotypes	Haplotype diversity (*h*)	Nucleotide diversity (*π*)	Mean number of pairwise differences (*k*)
North	NYS	2007.11	15	4	8	0.9048±0.1005	0.0036±0.0024	2.0190±1.2023
Yellow Sea								
Dongying	DY	2010.10	24	15	11	0.8478±0.0633	0.0041±0.0026	2.2536±1.2847
Yantai	YT	2007.11	23	13	13	0.8775±0.0607	0.0040±0.0025	2.2055±1.2641
Qingdao	QD	2009.07	22	17	13	0.8918±0.0550	0.0043±0.0027	2.3891±1.355
Haizhou Bay	HZB	2011.07	22	25	17	0.9740±0.0542	0.0067±0.0052	3.7186±1.9519
Shanghai	SH	2005.10	23	6	9	0.865+-9±0.0284	0.0030±0.0061	1.660±1.0126
Zhoushan	ZS	2009.05	8	4	4	0.7500±0.1391	0.0024±0.0019	1.3571±0.9330
Total	/	/	114	57	60	0.9650±0.0094	0.0047±0.0028	2.6267±1.4120

### DNA amplification and sequencing

The first hypervariable segment of control region was amplified via polymerase chain reaction (PCR) with universal primers of DL-S, 5’-CCCACCACTAACTCCCAAAGC-3’(forward), and DL-M, 5’-GCAACGTTCATATTCTCGGAGGC-3’(reverse). Reactions consisted of 10 × PCR buffer, 2.0mM MgCl_2_, 200μM of each dNTP, 0.3μM of each primer, 0.15 units of Taq DNA Polymerase (TaKaRa), and 1μL template DNA in a final volume of 25μL. PCR reaction was carried out in an Eppendorf thermal cycler with the following conditions: an initial denaturation for 5min at 94°C, followed by 35 cycles of 45s at 94°C, 45s at 50°C and 1min at 72°C, with a final extension at 72°C for 10min. The amplified products were gel separated and purified using the AxyPrep DNA Gel Extraction Kit. Purified products were sequenced using BigDye (Applied Biosystems, ABI) with both forward and reverse primers, and analyzed on the 3730 automated sequencer (ABI).

### Data analyses

Sequences were proofread, assembled and aligned using DNASTAR software (DNASTAR Inc., Madison, WI. USA). The 5’end of the control region fragment was extracted from the sequenced products by deleting partial sequences of the tRNA^Pro^ gene to prevent any bias in the estimates of sequence parameters from the control region. Molecular diversity indices such as haplotype diversity (*h*), nucleotide diversity (*π*), number of polymorphic sites (*S*), mean number of pairwise differences (*k*), transversions and transitions were obtained using the program ARLEQUIN Ver. 3.5 (Excoffier and Lischer, 2010).

Relationship among haplotypes was constructed using a neighbor-joining method performed in MEGA 5.0 (Tamura et al. 2011) with 1000 bootstrap replications based on distances calculated using the best selected model K2P. Minimum spanning trees of haplotypes were constructed under haplotype pairwise differences obtained in ARLEQUIN.

Genetic differentiation between pairs of population samples was evaluated with the pairwise fixation index *F_st_* (Excoffier et al. 1992). The significance of the *F_st_* was tested by 10000 permutations for each pair-wise comparison. Population subdivision and significant population structure was examined using a hierarchical analysis of molecular variance (AMOVA) with two groups: one group (NYS) and the other group (DY, YT, QD, HZB, SH, ZS).

The *D* test of Tajima (Tajima, 1989) and *Fs* test of Fu (Fu 1997) based on the infinite site model was calculated using ARLEQUIN. Tajima’s *D* and Fu’s *Fs* are very sensitive to population demographic expansions, which generally lead to large negative values. Historical demographic expansions were further investigated using the ‘mismatch distribution’ of all pairwise differences between mitochondrial sequences using ARLEQUIN. The time since population expansion was estimated using the equation *τ* = 2ut, where u is the mutation rate for the whole DNA sequence under study and t is the time since expansion.

Changes in effective population size (Ne) across time were inferred using Bayesian skyline analyses, which enable past demographic changes to be inferred from the current patterns of genetic diversity within a population (Drummond et al. 2005). BEAST v1.7 (Drummond et al. 2012) was used to create the Bayesian skyline plots with all populations. To test for convergence, we performed multiple analyses that were run for 10^8^ iterations with a burn-in of 10^7^ under the K2P model, a strict molecular clock and a stepwise skyline model. Genealogies and model parameters were sampled every 1 000 iterations. All operators were optimized automatically. Results of replicate runs for each lineage were pooled using LogCombiner, and the effective sample size (ESS) for each parameter exceeded 200.

## Results

### Sequence variation and genetic diversity

A 549bp segment of 5’ end of the control region was obtained for 137 individuals. The nucleotide composition of this segment exhibited high abundance in AT-content (34.92%+44.46%) and remarkable avoidance of G base (15.33%), which was consistent with the base composition of the most of fishes. Sequence comparison revealed 60 haplotypes that were defined by 57 polymorphic sites with 43 transitions and14 transversions (Genbank accession numbers: MF381080 -MF381139). Total 8 haplotypes were shared among individuals, among which one main common haplotype was respectively shared by 37 individuals (9 from DY, 8 from YT, 7 from QD, 3 from HZB, 6 from SH and 4 from ZS). The remaining 52 haplotypes were unique to their geographic populations. Intrapopulation diversity indices indicated that ZS population had the lowest genetic diversity among the seven populations. By contrast, the highest haplotype and nucleotide diversity was observed in HZB population. In summary, the seven populations of *J. grypotus* were mostly showed the high gene diversity and low nucleotide diversity. (Table 1)

### Population genetic structure

**Figure 2.**
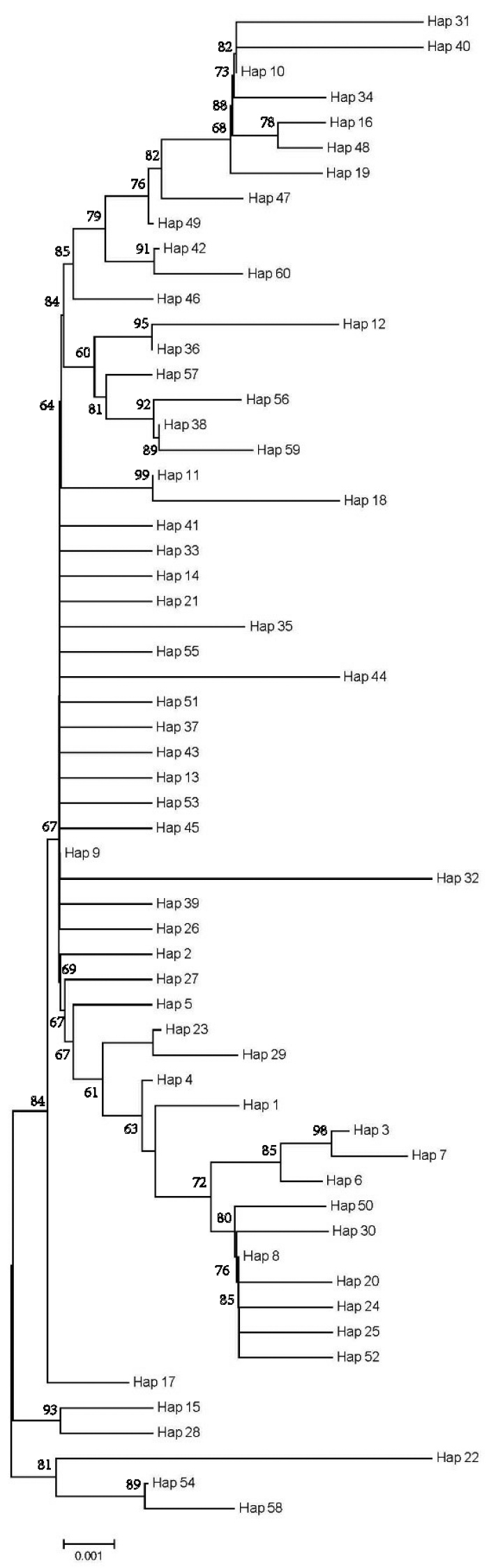
Phylogenetic tree of control region haplotypes constructed using neighbor-joining algorithms of *J. grypotus*.

**Figure 3.**
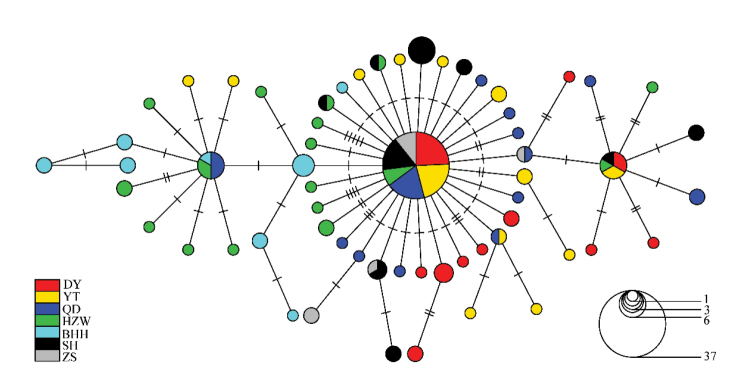
Minimum spanning network showing genetic relationship among mtDNA control region haplotypes in *J. grypotus* (Circles represent haplotypes with sizes proportional to their respective frequencies. Tick marks represent deduced numbers of nucleotide substitutions along each branch)

Unrooted phylogenetic tree was reconstructed by neighbor-joining analysis using 60 haplotypes with the best nucleotide substitution mode. There were no significant genealogical branches or clusters corresponding to sampling localities (Fig. 2). The minimum spanning network was generally star-like with one dominant haplotype shared by the most populations. (Fig. 3)

The pairwise *F_st_* values revealed that genetic differences between NYS and other populations were significant, and non-significant pairwise *F_st_* values were detected among other populations. (Table 2). The global (one gene pool) AMOVA showed only 4.58 % of the genetic variation was attributed to among populations. A hierarchical AMOVA showed that the proportion of covariance component and associated Φ statistics of the “populations among groups” were low and non-significant. This indicated that the genetic variation was mostly derived from “within populations” instead of from “among populations” or “among groups”. In general, the populations of *J. grypotus* were not behaved geographic genetic structure, despite that significant and low-to-middle genetic difference was detected between NYS and other population. (Table 3)

**Table 2.** Pairwise *Fst* (below diagonal) and associated *P* values (above diagonal) among populationsof *J. grypotus*

Population	NYS	DY	HZB	QD	SH	YT	ZS
NYS		0.00000	0.0024	0.0002	0.00000	0.0000	0.0004
DY	0.1252*		0.0566	0.0203	0.0009	0.0787	0.2426
HZB	0.0538*	0.0365		0.1550	0.0091	0.0334	0.3764
QD	0.0937*	0.0125	0.0120		0.0018	0.0786	0.5901
SH	0.1157*	0.0466	0.0405	0.0418		0.0035	0.1188
YT	0.1095*	-0.0004	0.0242	0.0031	0.0375		0.4208
ZS	0.1644*	0.0112	0.0619	0.0098	0.0521	0.0082	

**Table 3.** AMOVA of *J. grypotus* populations based on mtDNA control region sequences

Source of variation	Observed partition		Significance	
	Variance components	Percentage variation	Φ Statistics	P
1. One gene pool (DY, YT, NYS, QD, HZW, SH, ZS)				
Among populations	0.0212	4.58	Φ_ST_=0.046	0.00±0.00
Within populations	0.442	95.42		
2. Two gene pools (NYS) (DY, YT, QD, HZW, SH, ZS)				
Among groups	0.0032	0.7	Φ_CT_=0.007	0.200±0.00
Among populations within groups	0.198	4.27	Φ_SC_=0.042	0.000±0.00
Within populations	0.4421	95.03	Φ_ST_=0.0497	0.000±0.00

### Population historical demography

**Figure 4.**
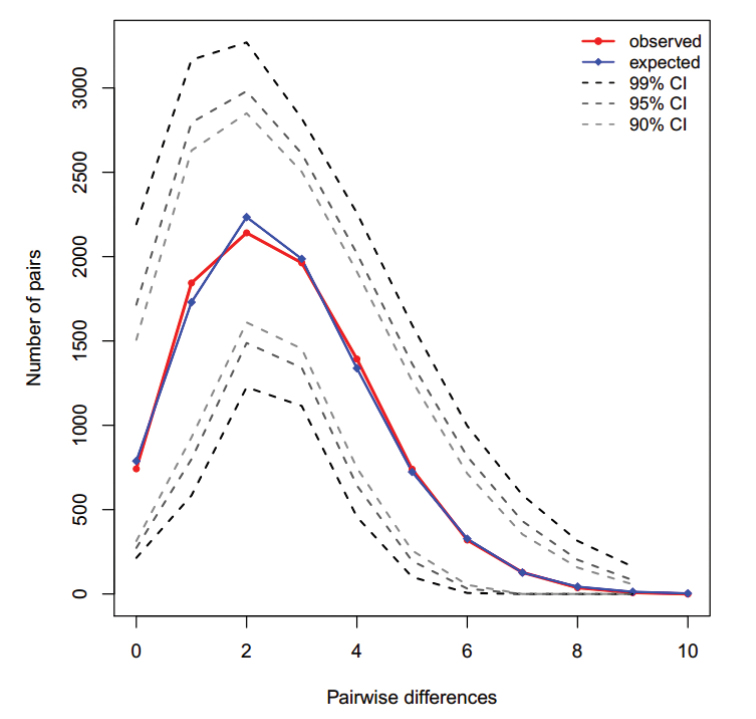
Observed and expected mismatch distribution under the sudden expansions model of the control region haplotypes in *J. grypotus*.

**Figure 5.**
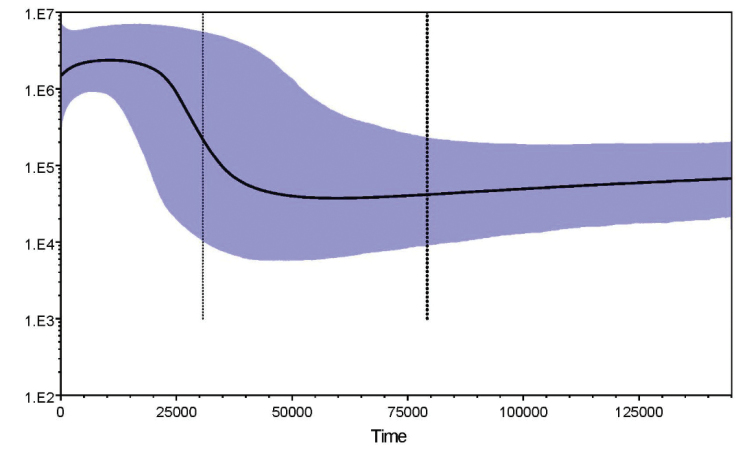
Bayesian skyline plots showing *N_ef_*T (*N_ef_*=effective female population size; T=generation time) changes through time in *J. grypotus* populations. Black lines are median estimates of *N_ef_*T; light lines represent the upper and lower 95% highest posterior density (HPD) limits of *N_ef_*T.

The results of neutrality test of all populations showed that Tajima’s *D* (*D*=-2.186; *P*<0.01) and *F*_S_ test (*F*_S_=-26.367, *P*<0.01) was negative and highly significant, meant departure from the selective neutrality. The sudden expansion model of mismatch distribution was unimodal and a valid goodness-of-fit was observed between observed and expected distributions (Fig. 4), indicating strong demographic expansion signals (Fig. 4). Based on *τ* values (*τ*=2.195) and divergence rate of 5%-10% per site per Myr (Brunner et al. 2001), the pure demographic expansion was about from 20,000 to 40,000 years ago. The timing of sudden expansions of *J. grypotus* populations dated to the last glacial period in the last Pleistocene. Bayesian skyline plot (BSP) suggested one episode of population rapid growth in *J. grypotus* populations. The time of expansions about mismatch distribution was consistent with the rapid population growth intervals generated by BSP (Fig. 5).

### Discussion

Mitochondria DNA, especially the control region, has the unique characteristics, such as maternal inheritance, high mutation rate (compared with nuclear DNA) and nonrecombinant DNA (Whitehead et al. 2003; Dowling et al. 2008). Therefore, it has been widely used in the researches of population genetic variation (Avise et al. 1986; Grant et al. 1998; Liu et al. 2006; Han et al. 2012; Song et al. 2013). In the study, the genetic diversity, population genetic structure and historical demography of *J. grypotus* were characterized using the first hypervariable region of control region.

### Genetic diversity

The genetic diversity of species is closely related to the adaptation for surroundings and their evolutionary potential, and has an important effect on species maintenance and conservation (Templeton, 2010). High level of genetic diversity usually possesses the strong capacity for environmental endurance and for population extension. In contrast, low genetic diversity generally tends to lead to population shrink, bottleneck and even extinction when facing the environment pressure. High gene diversity (*h*) suggests that *J. grypotus* populations have a strong adaptability of environment. However, the low nucleotide diversity (*π*), on the other hand, indicates that the populations have gone through bottleneck events and lost genetic information. The recent population expansion is likely to result in enhancing the retention of new mutations and a high genetic diversity that especially behaved in gene diversity because that the accumulation of nucleotide diversity was much slower (Grant et al. 1998). Some other fish in the Sciaenidae also show same phenomenon of “high *h* and low *π*” in control region, such as *Pennahia argentatus* (Han et al. 2008a), *Larimichthys polyactis* (Xiao et al. 2009), *Collichthys lucidus* (Song et al. 2013) and *Miichthys miiuy* (Xu et al. 2014).

### Population genetic structure

Population subdivision and genetic structure provides important proof for strategy of species protection in conservation genetics (Templeton, 2010). The phylogenetic and minimum spanning network displayed a shallow coalescence and non-significant genealogical branches or units. This was probably caused by the recent expansion after population bottleneck and (or) by the large gene flow among populations. This conclusion was sustained by the relatively low pairwise *F_st_* values among populations and non-significant among groups. *Pennahia argentatus*, *Nibea albiflora*, *Larimichthys polyactis* and *Miichthys miiuy* within Sciaenidae along the Chinese coastal waters perform the similar genetic structure pattern with *J. grypotus* (Han et al., 2008a; Han et al., 2008b; Xiao et al., 2009; Xu et al., 2014). Low genetic differentiation among *J. grypotus* populations further indicated the large dispersal potential and a large amount of migrants between populations. With spawning pelagic eggs and weakly migratory ability in juvenile and adult fish, the dispersal ability of *J. grypotus* appears to be passive transmission by environmental factors. The dispersal of pelagic eggs and larvae plays a fundamental role in the ecology and evolution of marine organisms (Kawabata et al. 2005; Faurby et al., 2012). Many marine organisms have pelagic eggs and larvae that can potentially interconnect distant populations through dispersal on ocean currents (Song et al. 2013; Puritz et al., 2017). The pelagic eggs and larvae could last for about 30 days, therefore the current patterns in the studied areas should facilitate the dispersal of *J. grypotus* pelagic eggs and larvae among populations.

The significant and low-to-middle genetic differences were behaved between NYS and other populations, which suggested that the deep sea area of the Yellow Sea might play the role of the geographical isolation. Although the distance between NYS and YT (or DY) popultions is quite nearly, the adult of *J. grypotus* could’t pass though the deep sea area of the Yellow Sea, duo to it prefers living, reproducing and migrating in the coastal waters (Zhu et al. 1963). The genetic connectivity for these populations would mainly rely on the dispersal of planktonic larvae, which is transported by coastal current. The coastal current from NYS to other populations would go through whole coastline of Bohai Sea, which would reduce the transmission efficiency and generate genetic differentiation.

### Population historical demography

Apartg from the life history and marine environmental factors, paleogeological changes and paleoclimactic fluctuations also have important influence on genetic diversity, genetic distribution pattern and effective population size (Hewitt et al. 1996). A series of large glacial-interglacial cycles caused pronounced fall and rise of the sea level in the late Pleistocene period, and glacial maxima at intervals of ~100,000 years over the past ~800,000 years were associated with declines in sea level of 120-140 m (Lambeck et al. 2002). This resulted in area and geologic structure dramatic changes of marginal seas of the Northwest Pacific (Wang, 1999). The living environment and habitats of fishes, such as *J. grypotus*, received the tremendous influence. With the drop of sea levels in the glacial period, *J. grypotus* could have incurred population shrinkage and preserved some genetic information. The star-like network with one dominant haplotype suggests that *J. grypotus* most likely spread from one glacial refugium.

Demographic and range expansion must generate the recruitment and habitat extension from the refugia during the interglacial period with the temperature increase, habitat enlargement and food richness. The same scenario also has been found in many studies about molecular phylogeography of marine fish, such as *Chelon haematocheilus* (Liu et al. 2007), *P. argentatus* (Han et al. 2008), *C. lucidus* (Song et al. 2013). Based on 5-10% divergence rate, the sudden expansion of *J. grypotus* was about 20-40 ka BP which reached in the Marine Isotope Stage 3 (MIS3, about 24-59 ka BP) interstadial in the last glaciation (Siddall et al. 2008). The elevated temperature that reduced global ice volume and sea-level rise brought in many rich resources and generated suitable breeding conditions for *J. grypotus* and subsequently led to rapid population growth and spatial expansion.

In conclusions, the glacial-interglacial cycles led to the climatic fluctuations in the Pleistocene have an important influence on abundance and distribution of *J. grypotus* populations. Early life-history characters suggest relatively strong dispersal potential by eggs and larvae for this species, which may play an important role in large gene flow among populations. Therefore, it is essential to strengthen the management of eggs and larvae protection in the approach of conservation strategy. With overexploited fishery resource and water eco-environment deterioration, *J. grypotus* and other traditional economic fish are severely threatened and depletion. The high level of genetic diversity of *J. grypotus* proves that it is not too late to increase awareness of protection. Our study will lay the foundation for periodic detection of genetic diversity, and provide scientific guidance for the management of fisheries and conservation efforts and for the sustainable development of *J. grypotus* resource.
